# Gender Differences and Lost Flexibility in Online Freelancing During the COVID-19 Pandemic

**DOI:** 10.3389/fsoc.2021.738024

**Published:** 2021-08-31

**Authors:** Michael Dunn, Isabel Munoz, Steve Sawyer

**Affiliations:** ^1^ Department of Management and Business, Skidmore College, Saratoga Springs, NY, United States; ^2^ School of Information Studies, Syracuse University, Syracuse, NY, United States

**Keywords:** online labor market, freelance work, precarity, COVID-19, gender, knowledge work

## Abstract

We report findings from an ongoing panel study of 68 U.S.-based online freelancers, focusing here on their experiences both pre- and in-pandemic. We see online freelancing as providing a window into one future of work: collaborative knowledge work that is paid by the project and mediated by a digital labor platform. The study’s purposive sampling provides for both empirical and conceptual insights into the occupational differences and career plans of freelance workers. The timing of the 2020 data collection provides insight into household changes as a result of the COVID-19 pandemic. Findings make clear these workers are facing diminished work flexibility and increased earning uncertainty. And, data show women are more likely than men to reduce working hours to help absorb the increased share of caregiving and other domestic responsibilities. This raises questions of online freelancing as a viable career path or sustainable source of work.

## Introduction

We provide empirical evidence and theorizing regarding how U.S.-based freelancers working on online platforms are responding to the coronavirus-induced crisis. To do this, we combine survey data with interviews of freelance workers who use one online work platform. We pursue this work recognizing that the implications to workers and labor markets, due the global COVID-19 pandemic, are profound (Atkeson, 2020; McKibbin and Fernando, 2020; Stephany et al., 2020; Wenham et al., 2020). We also see this work as providing a window into one possible future of work, online freelancing, that is both fundamentally precarious and an increasingly common form of contemporary labor (e.g., Erickson et al., 2019; Harmon and Silberman, 2019; Jarrahi et al., 2020).

Online freelancing reflects a form of labor market that relies on mediated and fully digital interactions between these workers and potential employers. Even as O’Farrell and Montagnier, (2019) articulate difficulties with determining the scale of platform-mediated work, estimates suggest there are 56 million online freelancers globally, 40% of whom are U.S. - based (Kässi and Lehdonvirta, 2018; Stephany et al., 2021). And, these online labor markets have grown by almost 50% since 2017 (Kässi and Lehdonvirta, 2018; Stephany et al., 2021). This growth reflects the lure of worker flexibility (finding work when one wants) aligning with desires of organizations for flexible workforces (finding workers as needed) (e.g., Kalleberg, 2003).

The global and virtual nature of online freelancing allows greater competition because of the low barriers to entry (Dunn, 2017). And, in countries like the U.S., online freelancers are independent contractors. This means they lack benefits like health care, retirement, leave, and other workplace protections afforded to full-time workers (International Labour Organization, 2016; McKay Pollack and Fitzpayne, 2019). This lack of labor protections means online labor markets and online freelancers are likely to feel the effects of economic changes such as the current pandemic more acutely than would those who have more labor market or workplace protections.

Further compounding the precariousness of online freelance work are the gendered realities and challenges for work-life balance, which are felt more acutely by women (Hannák et al., 2017; Ciolfi and Lockley, 2018). Women, for example, are responsible for 75% of unpaid care and domestic work around the globe (Moreira da Silva, 2019). As such, women are often engaging in additional work outside the traditional labor economy, what sociologists call a “second shift,” referring to unpaid household responsibilities (Hochschild and Machung, 1989), and a “third shift,” referring to unpaid emotional labor outside the home (Gerstel, 2000). Within the context of online freelancing, scholars have shown the naivete of the notion that these platform-mediated markets are gender-neutral (e.g., Foong, Vincent, Hecht and Gerber, 2018; Pichault and McKeown, 2019).

All of this drives us to pursue the following research questions: 1) What are the experiences and expectations of online freelance workers in the face of rapid market changes and the uncertainty of an ongoing global pandemic and 2) In what ways do the responses to the pandemic’s impact differ by gender?

## Why Workers Pursue Online Freelance Work

### Online Freelancing: Flexibility and Access to Work Online

Online freelancers are knowledge workers that find their work *via* online labor platforms. Because it is primarily cognitive work, this type of work can be done independent of location (Erickson and Jarrahi, 2016; Sutherland and Jarrahi, 2017). This market-mediated collaboration distinguishes online freelancing and online labor platforms like Upwork and Freelancer.com from gig-working sites like Uber or Deliveroo, as the latter involve physical on-site service delivery (Kalleberg and Dunn, 2016; Vallas and Schor, 2020). Likewise, online freelancing is distinct from micro-tasking sites like Amazon’s Mechanical Turk (AMT) by the complexity of the tasks and the expectation of worker/employer interactions for guidance and sense-making (Wood et al., 2019a; Gray and Suri, 2019; Howcroft and Bergvall-Kåreborn, 2019).

One of the most-often-noted reasons by people pursuing work online is the flexibility it affords (Malone, 2004; Horton, 2010; Johns and Gratton, 2013; Kuek et al., 2015; Sundararajan, 2016; Wheatly, 2017). Early accounts highlighted that online work allowed the flexibility to care for home, school or business responsibilities while earning a salary (Kuek et al., 2015). Recent accounts are more critical of working online, noting flexibility also often means that access to work is uncertain and other options may no longer be available given changes in the labor markets (e.g. Aroles et al., 2019; Wood et al., 2019b; Pichault and McKeown, 2019).

To better understand flexibility in online freelance work, we draw insight from other technology-enabled flexible working arrangements such as telework, telecommuting, nomadic, flextime, and flexplace: all important topics of scholarship since the 1990s (see Baltes et al., 1999; Sutherland and Jarrahi, 2017; Erickson et al., 2019). Building from these areas, scholars have identified potential advantages to flexible scheduling such as reducing work-family conflict (Shockley and Allen, 2007); time and location independence (Erickson and Jarrahi, 2016; Sutherland and Jarrahi, 2017; Erickson et al., 2019); and, allowing paid work to be combined with life circumstances that prevent regular work (Silver and Coldschejder, 1994).

Yet, studies on the value of flexible scheduling show mixed results, calling into question what exactly is meant by flexible scheduling (e.g., Baltes et al., 1999; Shockley and Allen, 2007). Scholars have begun to distinguish worker-controlled flexible scheduling from manager-controlled flexible scheduling (Henly et al., 2006). Many of the potential advantages of flexible scheduling are associated with worker-controlled flexibility. Manager-controlled flexibility is associated with the opposite--because from the worker’s point of view it creates uncertainty and inhibits planning (Hyman et al., 2005; Lambert et al., 2012).

The boundary between worker-controlled and manager-controlled flexibility is ambiguous as the practices of negotiating working times are bound up in workplace power relations (Lambert et al., 2012; Wood, 2016). For instance, Wood (2016) supermarket workers were formally free to declare the hours that they were available to work. In practice, however, they had to accept disruptive shifts or risk no longer being offered work. Likewise, the freelance technical contractors studied by Barley and Kunda (2011) were formally free to set their own working hours, yet, many worked through evenings and holidays because they believed this would decrease their chances of being let go and increase chances of future contracts (see also Fraser and Gold, 2001; Gold and Mustafa, 2013). Similarly, Shevchuk et al. (2019) report that Russian freelancers with high autonomy and flexibility in scheduling still choose to work long and nonstandard hours, to the detriment of their wellbeing. Most recently, Shevchuk et al. (2021) found that the online labor platform’s always-on presence constrains freelancers’ time, such that workers with clients in different time zones take on hours that are more nonstandard.

Thus, while the early literature on flexible scheduling framed flexibility as a matter of freedom from formal constraints, more recent literature highlights structural factors that constrain workers’ ability to manage their time, with the freelancer less able to control their schedule. Lehdonvirta (2018) study of online gig workers found that a worker’s ability to schedule their work was ultimately determined by two factors: the availability of gigs and the worker’s dependence on income from the gigs. If gigs are scarce, and the worker depends on income from gigs, the worker has to remain constantly on call to be ready to sign up for gigs as soon as they become available (Wood et al., 2019b). If gigs are plentiful, and the worker sees the income as extra, they will take jobs of interest and as it suites them (high flexibility).

### Gendered Nature of Online Freelance Work

Online freelance work is also gendered work (Czarniawska, 2014; Ciolfi and Lockley, 2018). From at least the 1960s, scholars have been examining the working women’s perspective by looking at the broader context in which they do paid and unpaid work, including the relationship of work in the public and private sphere (Hochschild and Machung, 1989; Gerstel, 2000; Morini, 2007; Balka and Wagner, 2020). Scholars have shown ways in which work and non-work times and effort are interwoven, with boundaries gradually blurring for those with flexible arrangements (Ciolfi and Lockley, 2018; Cousins and Robey, 2015; Erickson, et al., 2019; Humphry, 2014). Analyzing the role of gender in emerging forms of work seems even more relevant given that women have historically assumed a greater share of domestic responsibilities (Barulescu and Bidwell, 2013; Blau and Kahn, 2016; Wiswall and Zafar, 2018).

Scholars are now calling for more research on the working experiences of women and other marginalized and underrepresented groups (such as racial and ethnic minorities) (Bardzell, 2010; Balka and Wagner, 2020). Balka and Wagner (2020) note that while scholarship has produced a rich body of work in many domains, including mobile or nomadic work, “These new forms of work emerge in environments in which gender-based differences in home life are likely to reinforce gender-based differences in the workplace, and historical conceptions of skill associated with (male) gender may gain foothold” (p. 44). Hannák et al. (2017) found that perceived gender and race could have significant negative consequences for individual’s search ranking as existing biases can manifest in online labor markets.

Additionally, Ciolfi and Lockley (2018) highlight that knowledge workers in work from home arrangements face changes and challenges in setting work-life boundaries. Both males and females face challenges managing their workload, prioritizing work versus family, and setting boundaries to take family and personal time. Collins et al. (2020) examined the gender gap among teleworkers in the face of the COVID-19 pandemic, given “... It is possible that the increased prevalence of telecommuting may facilitate greater equality in task-sharing and the gendered division of (labor) at home.” (p. 2). Contrastingly, the authors found the pandemic has increased gender inequality in the online labor force. Mothers were more likely to reduce their working hours than fathers, with mothers of children under 13 years old facing the largest reduction in hours, even as fathers’ work hours remained largely stable. Similarly, Kalenkoski and Pabilonia (2020) found that following the first wave of COVID-19, women with younger children felt greater impacts on their working time than did single men.

## Methods

### The Panel Study

To pursue the study’s research questions, we draw data from an ongoing panel study of 68 U.S.-based freelance workers who seek work via the online labor platform Upwork (see upwork.com), the dominant online job-seeking site and gig-work platform that specializes in knowledge-based work. Of these 68, 38 participants spoke with us before, and 30 spoke with us after mid-March 2020, when we began asking how they were faring in the face of the COVID-19 pandemic (see Table 1 for summary statistics). The analysis reported on here builds from interview transcripts, field notes, and survey data.

**TABLE 1:** Summary Statistics.

Job Classification		Gender	
Administrative	34%	Female	60%
Technology	30%	Male	40%
Creative	36%		
**Role of Freelance Work**		**Length on Digital Platforms (in years)**	
Primary Source of Employment	Additional Source of Employment	Min	0
53%	47%	1st Qu.	2
		Median	3
		Mean	5.246753247
		3rd Qu.	8.5
		Max	20
		NA’s	0

### Data Collection

The panel study builds from purposive sampling of workers who pursue freelance work as a primary or secondary source of income, selected from one digital labor platform, Upwork, to minimize platform effects. The sample reflects a range of work types, skill levels, online experience, gender, race, and success online. Workers were selected across three broad categories of occupations–creative, administrative, technology. Years working on platform was used as proxy for online experience. Our gender breakdown is representative of the market, slightly skewed female (The Freelance Creative, 2016). Participants were hired through the platform and paid as they would for any job found on Upwork. Once hired, participants spent 15 min completing a survey that provided us basic demographic information and an overview of working arrangements and experiences. Once the survey was completed, the participant was interviewed via a recorded line by a research staff. The surveys were then transcribed in preparation for analysis.

As is typical in inductive and exploratory work, the interview, survey and secondary data were subject to iterative analyses, and this was used to guide incremental changes to both the survey and interview guide. The initial survey and interview guide were designed to complement one-another, including a set of questions that drove the direction of the secondary data analysis. The study’s longitudinal design and interview schedule provided us time to conduct interim analysis of the data and to reflect on changes in understanding and - importantly - allowed for the inclusion of COVID-19 related data collection.

### Data Analysis

Interview and survey data were analyzed independently and then together with the secondary data. Interviews were transcribed and, in conjunction with detailed interview notes, were inductively coded, guided by the stated research questions. We then used a thematic analysis to illuminate patterns and common themes within the interview data. Initially, researchers individually reviewed all interview transcripts and developed reports on common patterns. Themes were shared in a group channel and discussed during weekly meetings, with careful note-taking and additional reading to develop a documented understanding of these themes and the data supporting them. Themes were then organized and interrogated within the context of our stated research questions. These findings are useful to the extent they provide greater insight than do rival explanations.

## Findings

Data show online freelancers in our study are experiencing changes in their working arrangements. We focus on two insights relative to the first research questions: diminished work flexibility and increased earning uncertainty. We also report on the differential effects women freelance workers are experiencing, responding to our second research question. Broadly, our work aligns with previous studies showing that online freelance work is precarious, with the COVID-19 pandemic exacerbating this precariousness.

### Diminished Work Flexibility and Increased Earning Uncertainty

Relative to the first research question regarding experiences and expectations of online freelancers grappling with the pandemic, data show there is decreased worker-controlled flexibility, as freelancers are being driven by a desperation to secure work that is rooted in the acknowledged precarity of their situation. This is true for both long-term and new freelancers, and for both full- and part-time freelancers. Freelancers indicated they faced significantly more competition, resulting in both fewer proposals accepted and lower compensation. An experienced female freelancer reflects: “*I think more people are trying to find online work because they’re out of their normal jobs [...] I think there’s a lot less work to go around than usual because everyone is scrambling to make money, either while they are at home because they can’t go into their office, or while they’re laid off [...] So this current state definitely makes it more difficult” (FPAC040720201)*.

Some freelancers reported that the new jobs and tasks being posted also reflect lower rates, leading to a sense that clients are taking advantage of workers during the pandemic: “*[...] there is going to be a lot of taking advantage of workers and their need to put food on the table” (FPAC042620201)*. Additionally, stable long-term clients who provided a dependable source of income are stopping current projects and not requesting new work: “*The two (clients) that I lost due to this virus were long-term. One of them I had been working with for approximately a year and the other one was several months, but I don’t know what’s going to go on with them, if they’re even going to come back or anything” (FPAC042520202)*.

We also found that freelancers who previously relied on freelance work as a secondary source of income have become more reliant on platform-based work due to the ongoing COVID-19-related business closures (Kochhar, 2020). One participant noted: “*My main business has basically dropped off. So I’ve had to really up my freelance gig work…. All my streams of income came to a stop which put me more online doing freelance work*” *(FPAC042620201)*. Combined, these findings suggest that freelancers on Upwork are witnessing their worker-controlled flexibility diminish in the face of the pandemic (in the U.S.).

### Gender Magnifies the Loss of Flexibility and Rise of Uncertainty

Women freelancers in our panel were more likely to report reduced flexibility, increased uncertainty, and substantial changes to their working routines. To this point, one female panelist noted: “*The other thing that has affected me is that my kids are home now, so I’m having to homeschool my two children on top of trying to stay productive and earn income for myself, so I’m definitely feeling it” (FPAC04222020).*

Data show women were more likely to report having unpredictable earnings from freelance work. And since the pandemic started, the average number of freelancing hours worked decreased for respondents, with women’s reported weekly working hours dropping at nearly double the number of men’s (12.7-h and 6.5-h decrease respectively). Increased home responsibilities left some women with less time to find and secure work. “*It feels a little slower than before. I am also a full-time mom, so my kids are staying home all the time now because the schools are closed. I don’t have as much time to look for work as I did.*” *(FPCC04142020).* Our data reflect what others report: gendered differences in responsibility appear to be magnified in households with children because of COVID-19-induced lockdowns and school closures (Collins et al., 2020; Reichelt et al., 2020).

This insight must be situated in the reality that research continually re-establishes women bear a greater share of domestic responsibilities (Hochschild and Machung, 1989; Gerstel, 2000; Barulescu and Bidwell, 2013; Blau and Kahn, 2016; Wiswall and Zafar, 2018). The Institute for Fiscal Studies (2020) reports mothers doing paid work at home are more likely than fathers to be spending their work hours trying to care for children. Mothers are able to do 1 h of uninterrupted work for every 3 h done by fathers. In addition, mothers take on more chores and spend more time with children in homes where there is both a working mother and father (Institute for Fiscal Studies, 2020). Likewise, mothers are more likely than fathers to have left paid work and experienced a larger reduction in their hours. And, these findings are amplified in single-parent families with female heads of households. Based on our interviews, women are more likely than men to discuss their work and schedule with relation to their home and family lives. For example, women spoke of keywords such as “kid/kids,” “child,” “baby,” “son,” and “daughter” at higher rates than did men.

These findings align with existing research on the gendered nature of work. Foong et al. (2018) also report that women across the entirety of the Upwork platform earn less per hour than the median man on Upwork, even when controlling for key variables such as work experience, highest education level, and job category. And, while it is beyond the scope of our exploratory study, our findings contribute to broader discourse on precarity and the feminization of cognitive labor (per Morini, 2007).

## Discussion

Findings focus on the impact of COVID-19-induced changes to the working arrangements of U.S. online freelance workers, providing insight into these new forms of labor and markets. Data illuminate differences between the realities of online freelance work and assumptions about both project-based work and online labor markets. One of the most basic assumptions, the premise of flexibility - a core reason for pursuing online freelance work, is challenged by these data. Findings show that flexibility for pursuing work is constrained by changes in people’s household arrangements. And, the flexibility to select work that aligns with one’s interests and schedules is being challenged by the whipsaw changes in the competition for online work, magnified by the increase in the number of people seeking work online (Stephany et al., 2020).

While inconclusive, given the exploratory nature of this work and the small number of participants, our data show there are decreases in weekly earnings and significantly greater difficulty in securing work since the start of the pandemic. These data also make clear that these earning losses vary by occupation. While occupational differences were not the primary focus of this study, we see the need for future research to explore these differences. For example, participants pursuing work in creative occupations such as marketing, design and writing reported experiencing precarity more often than did those in other occupational classes. Creative workers also reported a greater dependency on the wages from online and gig work while reporting the lowest access to health benefits. These workers also reported greater unpredictability of weekly earnings and experienced the largest drop in working hours when comparing post-COVID-19 data to what had been happening.

In the U.S., freelancers’ circumstances are further exacerbated by the under-regulated nature of both contingent labor and the market-making power of digital labor platforms. For example, freelancers in the U.S. are typically not eligible for unemployment, lack access to employer-provided benefits like healthcare and to labor protections provided full-time employees. Berdahl and Moriya (2021) looked at insurance coverage across all forms of non-standard work and found freelancers as the least insured of all non-standard workers. Given our data is based in a virus-centered pandemic, these data make clear the magnitude of freelancers’ precarity.

Our interviewees also reported significant decreases in the hours worked (across all occupations). We surmise the decrease may be explained by the realities of freelancers having to re-balance their household and non-work lives. This includes accommodating changing work and non-work boundaries and arrangements, with spouses and children who are home from school now competing for time and space in the household. And for our interviewees, the effects of re-configured family arrangements had a greater effect for women freelancers’ ability to do work in at least three ways. First, evidence shows that women are more likely to be pursuing freelance work - in part because of the flexibility this provides them. Second, occupations that are gendered outside of the digital labor platforms, remain gendered on these online platforms. Third, gender is visible in the profiles, experiences, and comments on these platforms (e.g., a client leaving a positive comment such as “She was incredibly responsive” leads with gender).

## Conclusion

Combining survey and interview data, the work reported here provides unique empirical and nascent conceptual insights into some of the impacts of COVID-19 on the online labor market and experiences of online freelancers in the U.S. Taken together, findings suggest that the concept of work flexibility, one of the primary reasons for pursuing online freelancing, might be better understood in the context of this pandemic and market shock as work desperation. Motivated by flexibility, freelance workers pursued online work that fit with household and non-work arrangements. Online work is always precarious, as our data and findings from others make clear. But, this precarity seemed a reasonable risk to preserve flexibility, in pre-pandemic times. That is, these online workers adjusted to their risk and found ways to make it work for them. Market shocks change the calculation in ways that seem to overwhelm these plans, leading workers to eschew flexibility as they scramble for work, even in the face of less flexible household arrangements and more demands. Facing increased competition for work online, freelancers will have to bid for more work and accept lower wages, even when this strategy is not in their best interest.

Some scholars assert that online labor markets are effectively eliminating the known gender inequalities found in the traditional labor markets (e.g., Gomez-Herrera, and Müller-Langer, 2019). Our findings suggest the pandemic has disproportionately impacted female online freelancers. This is an unfortunate but repeated finding across the entire labor market spectrum (e.g. Office of Congresswoman Katie Porter, 2020; Institute for Fiscal Studies, 2020; Chung and Tanja van der Lippe, 2020). For women doing technologically-mediated work remotely, the strongly gendered societal norms of household responsibilities means that women are bearing the brunt of the pandemic downturn. The implication is that the present economic travail is not only reversing decades of progress in gender equality; it is exacerbating disparities with the distribution of familial responsibilities. Simply, female online freelancers (and female workers in general) are facing a “double disruption” to their working lives and two generations of progress stands to be lost.

## Data Availability

Data are part of an ongoing longitudinal study and are not sharable at this time. Requests to assess the data should be directed to mdunn@skidmore.edu.
